# Make me a match: Creating research partnerships to build capacity for the evaluation of community initiatives

**DOI:** 10.1017/cts.2025.10068

**Published:** 2025-06-03

**Authors:** Christine M. Weston, Kristina Weeks, May Lynn Tan, Lee Bone, Jill Marsteller, Albert W. Wu

**Affiliations:** 1 Johns Hopkins Bloomberg School of Public Health, Baltimore, USA; 2 Johns Hopkins University School of Medicine, Baltimore, USA; 3 Johns Hopkins Medicine Armstrong Institute for Patient Safety and Quality, Baltimore, USA; 4 California Association of Food Banks, Oakland, USA

**Keywords:** Community-engaged research, community-academic partnerships, program evaluation, matching service, collaboration

## Abstract

**Introduction:**

While organizations leading community initiatives play a crucial role in tackling public health challenges, their difficulties in designing rigorous evaluations often undermine the strength of their proposals and diminish their chances of securing funding. We developed a matching service funded by the Robert Wood Johnson Foundation’s Evidence for Action program to bridge these gaps. This service identified matched applicants involved in community-engaged research with evaluation experts to provide complementary expertise, strengthen evaluation capacity, and enhance participants’ ability to secure funding.

**Methods:**

We conducted a mixed-methods evaluation of the pilot phase of the Accelerating Collaborations for Evaluation Matching Service from August 2018 to February 2021. Data sources included program records, participant surveys administered at 3-, 6-, and 12-months post-match, and semi-structured interviews conducted at 12–18 months post-match. We assessed outcomes such as match success, resubmissions, funding rates, and participant satisfaction.

**Results:**

Over the 2.5-year pilot period, the matching service successfully matched 20 of 24 referred applicants. Among these, 50% submitted revised proposals, and a third of secured funding. Survey results indicated widespread satisfaction with the partnerships. One-year interviews highlighted complementary expertise, bidirectional learning, and capacity-building as key benefits of these partnerships.

**Conclusion:**

This pilot demonstrated the feasibility, acceptability, and impact of the matching service in creating rewarding collaborations for community-engaged researchers. Beyond funding outcomes, participants uniformly valued the partnerships and described them as mutually satisfying. This model offers a scalable approach to creating research partnerships to build capacity for the evaluation of community initiatives.

## Introduction

Community-engaged research (CEnR) is pivotal to advancing population health and health equity. Yet the value of such work cannot be demonstrated without rigorous evaluation. Many community-based organizations that address complex public health problems lack sufficient evaluation capacity – whether methodological expertise, dedicated staff, or financial resources – and therefore struggle to design robust evaluations for the competitive grant proposals funders now require. Recognition of this gap has spurred a growing literature on strategies to build and sustain evaluation skills within community settings [1–8]. This article describes a unique matching service designed to connect community investigators with experienced evaluators to build capacity for the evaluation of community initiatives.

In 2014, the Robert Wood Johnson Foundation (RWJF) unveiled its Culture of Health Action Framework to guide its funding priorities [9–17]. As part of this vision, it launched Evidence for Action (E4A), which funds research that evaluates the impact of policies, programs, or practices on health outcomes and generates evidence to inform policy and practice.

E4A distinguishes itself from other grant funding mechanisms in two significant ways: (1) it accepts research proposals on a rolling basis, and (2) it actively encourages applications from academic and nonacademic organizations, including nonprofits, social service agencies, and government entities. During the review process, E4A identified a recurring challenge: many proposals lacked robust evaluation designs capable of supporting causal inference – a critical funding criterion needed to conclusively link outcomes to the interventions being studied. Another common problem faced by E4A applicants was the ability to select realistic effect sizes to inform calculations power, sample size, and minimum detectable effect [18]. These methodological challenges often prevented E4A from funding projects that otherwise aligned with its priorities – especially from nonacademic organizations that had less experience with rigorous research designs – and potentially led to missed opportunities to support the broader adoption and scaling of promising public health initiatives.

Recognizing this gap, E4A saw an opportunity to support applicants by pairing them with researchers with extensive evaluation expertise to help them strengthen their evaluation designs, enhance the quality of their research proposals, and increase their chances of securing funding. Thus, in 2018, E4A awarded a grant to the Johns Hopkins Bloomberg School of Public Health (JHBSPH) to establish the Accelerating Collaborations for Evaluation (ACE) Matching Service. This initiative aimed to create partnerships between a selection of applicants to E4A and experienced researchers who could provide expertise to strengthen and enhance the fundability of their research proposals. This paper explores the processes and outcomes of applications (called “cases”) referred to as the ACE Matching Service from August 2018 to February 2021. This service remains active as of the time of this publication.

### The accelerating collaboration for evaluation (ACE) matching service

The ACE Matching Service was led by two professors in the Department of Health Policy and Management of the JHBSPH with expertise in health services research, health policy, implementation science, CEnR, and health equity research, and supported by a senior advisor with over 40 years of experience in community-based participatory research (CBPR). Additionally, four researchers served as project acceleration liaisons (PALs), acting as the primary contact persons for E4A applicants referred to the matching service. Each case was assigned a lead PAL, but the PALs collaborated closely on all cases. The ACE Team also partnered with an E4A liaison to develop and refine the program, holding bi-weekly meetings to discuss referred cases, establish evaluation metrics, define parameters for seed funding, and address challenges that arose during the pilot project.

PALs were selected from university faculty, staff, and doctoral students as individuals with strong interpersonal and communication skills, a dedication to working with community organizations, and/or strong knowledge of evaluation methods. Two of the PALs were experienced researchers with comprehensive training in public health, including CBPR, health services and outcomes research, health policy, evaluation methods, and implementation science. One PAL was an advanced doctoral student in the Department of Health Policy and Management, and the other was a senior research program manager for health equity research programs with an MS in Public Health. The level of effort dedicated to the pilot project varied among the PALs and fluctuated over time, depending on the needs of the project at any given stage, with PALs contributing approximately 20%–30% of their time to the program. All PALs were supported through grant funding.

The two-year pilot study (and NCE) of the ACE matching service was funded by a grant from the RWJF, which supported the program’s design, implementation, and evaluation. The $280,000 annual grant covered start-up expenses, administrative overhead, and salaries for the principal investigators, a senior faculty advisor, four PALs, a project coordinator, and an administrative assistant, along with an additional $200,000 for seed funding and gift card incentives for survey participants. Future implementation costs will vary depending on staffing and the lead organization, but a replication of this model would likely incur lower costs due to reduced design and evaluation planning needs. As such, the pilot’s costs may not reflect the funding required for future versions of the program.

Traditionally, the E4A program used a two-stage application process. In the first stage, applicants submitted a two-page letter of intent (LOI), which could be submitted on a rolling basis. The selection committee reviewed the LOIs and either issued a turndown decision or invited the applicant to submit a full proposal. The introduction of technical assistance (TA) created a third pathway for funding consideration. A subset of applicants, identified by E4A during the review process as potentially benefiting from TA, were offered the chance to discuss their proposal’s strengths and weaknesses with an E4A team member. If both parties agreed that the project would benefit from the addition of a researcher to their team, the applicants were invited to participate in the ACE Matching Service and encouraged to revise and resubmit their proposal after going through the matching process. Nearly all applicants, who were offered, TA accepted the opportunity to discuss their proposals with E4A, and all applicants, whose projects were deemed a fit for matching, were then referred to ACE. E4A did not share with ACE how many applicants declined TA overall.

In this paper, we refer to the individual who submitted an LOI to the E4A program as the “applicant” and the individual matched with the applicant as the “researcher.” Once the match is made, this relationship is called the “partnership.”

**Figure 1.**
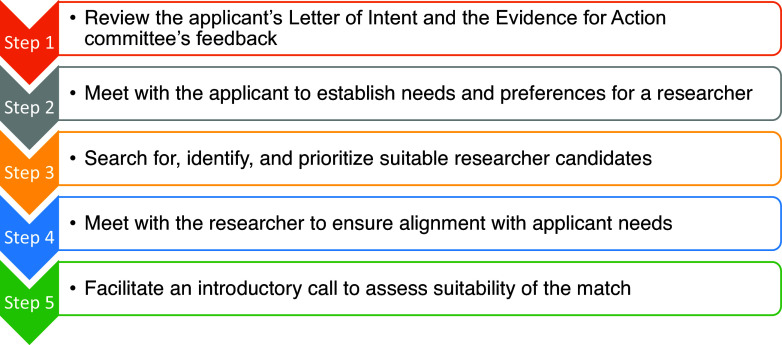
The accelerating collaborations for evaluation (ACE) matching process.

The matching service process was conducted in five steps (Figure 1):

The first step in the matching process required the assigned PAL to develop a clear understanding of who the applicant was, what they were proposing, and why they had been referred to the matching service. This step involved reviewing several documents, including (1) the applicant’s CV, (2) their organization’s website, (3) the proposed intervention and evaluation design from the LOI, and (4) the E4A liaison’s summary of the committee feedback highlighting weaknesses or areas needing further elaboration, clarification, or improvement. E4A provided all of this information directly to the PAL when the case was referred to the matching service.

Next, the PAL called the applicant to explain the matching service, discuss their research proposal, and assess their needs and preferences for a research partner to address the evaluation plan and design. Needs were broadly defined and varied widely, including specific subject matter expertise, methodological skills, familiarity with specific target populations (e.g., school-aged children), experience with particular interventions (e.g., educational programs), or with specific health outcomes (e.g., weight loss or Hemoglobin A1(c). Applicants also expressed preferences, such as a desire to be in close geographic proximity to the research partner, having shared lived experiences (e.g., race or gender) with their research partner, or being paired with a research partner with a track record of conducting CEnR. The PAL also asked if the applicant had any researchers in mind for consideration.

Once the applicant’s criteria were established, the PAL searched for an appropriate match. This process included reviewing applicant suggestions, consulting the ACE team for recommendations from their professional networks, and conducting literature searches to identify experts in the field. Potential researchers were ranked based on their fit with the applicant’s needs and preferences. There was no formal, one-size-fits-all system for ranking research partner candidates. Instead, we used an intentionally flexible process to accommodate the diverse needs and priorities of each applicant. PALs developed their own criteria-based approaches, first identifying the factors most important to the applicant. When helpful, they used tables to track candidate alignment with these priorities, giving preference to those most closely aligned with the applicant’s needs. When a top candidate was selected, the PAL contacted them via email to request a meeting.

The PAL then met virtually with the identified researcher to introduce the matching service, provide an overview of the applicant’s project, and assess the researcher’s expertise, interest, and availability. If the researcher was deemed a suitable match, they were introduced to the applicant. If not, the PAL moved to the next researcher on the list, repeating the process until a match was found.

The final step involved a facilitated introductory call between the applicant and the researcher. This meeting provided an opportunity to discuss the project and determine if the match was mutually agreeable. If both parties confirmed the match, it was finalized. If not, the PAL and applicant returned to the list to identify the next preferred candidate.

### Seed funding

A key and distinctive feature of the matching service was the provision of seed funding to compensate both the applicant and the researcher for their time and effort in developing the partnership and revising the LOI for resubmission. Acceptable expenses included time spent individually or collaboratively working on the revised LOI, obtaining pilot data, resources for analytic support, and travel for in-person meetings (discontinued at the onset of the COVID-19 pandemic).

### Design consultation

Partnerships were also offered opportunities called “design consultations,” facilitated by our E4A liaison. The purpose of these consultations was to help partnerships better align their study designs with E4A selection criteria by considering ways to enhance the rigor, feasibility, and potential impact of their proposed research, such as through modifications to the research approach or methods.

The matching process required extensive interaction between the PAL and the applicants. PALs played multiple roles, serving as educators, consultants, advocates, and liaisons between E4A and the partnerships. They guided applicants in understanding E4A’s processes, expectations for LOI revisions, and selection criteria. PALs dedicated significant effort to identifying suitable matches, conducting literature searches, gathering feedback from team members and members of their networks, evaluating options, and narrowing the list. After a match was made and a partnership formed, PALs assisted with seed funding applications, coordinated design consultations, and monitored the partnership’s progress toward resubmission to E4A or other potential funders.

## Methods

This evaluation used a mixed-methods evaluation to assess the ACE Matching Service processes and outcomes. Data were collected from program records, surveys at three time points (3 months, 6 months, and 12 months), and semi-structured interviews conducted 12–18 months after matches were formed.

The first goal of this project was to successfully match each applicant with a research partner. We aimed to achieve a successful match on the first attempt. The second goal was for the applicant and researcher to work together to revise and resubmit their LOI to E4A.

We collected process, intermediate, and outcome measures to evaluate the effectiveness of the matching service. Process measures included (1) the percentage of referred applicants matched with a researcher and (2) the time it took to make the match). Intermediate measures were (1) the percent of partnerships that submitted a revised LOI to either (a) E4A or (b) another funder, and (2) the time from the initiation of the partnership to the LOI resubmission). The primary outcome measures were (1) the percentage of revised LOIs submitted to E4A that resulted in an invitation to submit a full proposal and (2) the percentage of revised proposals that received funding. Additional outcomes of interest included the percentage of partnerships that applied for seed funding (and the average amount of seed funding per partnership), overall satisfaction with the match, and overall satisfaction with the matching service.

We sent a brief online survey to each member of the partnership three months, six months, and one year after the start of the partnership. The 3-month survey contained 13 multiple-choice questions that asked participants about collaboration with their partner, communication with the PAL, satisfaction with the matching service, intention to submit a revised LOI, and perceptions of the seed funding on a 5-point Likert scale. The survey also asked participants to respond to the following five questions: (1) Do you consider your partnership successful? (2) How are you (or your organization) benefiting from this partnership? (3) What challenges have you encountered (if any), and how have you addressed them? (4) Could we do anything further to help support your collaboration/partnership? (5) Do you have any other comments or suggestions for us about how to improve this service?

The 6-month (check-in) survey included 3 of the 13 questions that were asked during the 3-month survey, namely, “The partner I was matched with was an appropriate collaborator for me;” “I am pleased with the way the collaboration is proceeding;” and “Overall, I am satisfied with the assistance that I have received from the ACE Matching Service.”

The one-year follow-up survey asked participants to report their overall satisfaction with their partner, the design consultation they received, and the matching service.

Between 12 and 18 months after the match was made, we asked participants to participate in a one-hour semi-structured interview about their experience with the ACE Matching Service. The five interview questions were: (1) In what ways did you or your organization benefit from this partnership? (2) What do you think helped to move this partnership forward the most? (3) How would you define the success of the partnership? (4) How helpful was the seed funding for developing your partnership? (5) What challenges or barriers did you face during the partnership? and (6) What are your overall impressions of the matching service? Participants were interviewed by a team member other than their primary PAL contact person.

## Results

### Participant organizational affiliation and geographical location

During the pilot period of the ACE Matching Service, E4A referred 24 cases to the matching service. Of the 24 E4A applicants included in the pilot, 12 (50.0%) were from community-based, nonprofit, or nongovernmental organizations, 33.3% worked at academic institutions, and 16.7% worked at government/public sector agencies or organizations. Among these applicants, 3 were from the Northeast, 3 were from the Midwest, 7 were from the West, and 11 were from the Southern US states.

Of the 18 researchers matched with an applicant, 16 (88.9%) were affiliated with academic institutions, 1 was affiliated with a research/evaluation firm, and 1 was affiliated with a government/public sector agency. Two researchers were from the South, four were from the Midwest, six were from the West, and another six were from the Northeast.

### Primary outcomes measures

Of the 24 cases referred to ACE, 4 of these cases were not ready to be matched due to COVID-19-related constraints. Of the 20 cases that were ready to be matched, 100% of the cases were matched successfully. The matchmaking process ranged from less than a month to seven months, with a mean of 2.7 months.

In all but two cases, applicants were highly satisfied with the first researcher with whom they were matched. In one case, an applicant needed to be re-matched with a new research partner because the first researcher was overwhelmed with professional and personal stress due to the COVID-19 pandemic and did not have the time to devote to the partnership or the LOI revision. In the other case, after a few meetings, the applicant informed us that she did not feel that her partner was a good match for her after all, but did not elaborate on the reasons. She ultimately was matched with a new research partner, with whom she was able to secure funding for her project.

Among the 20 matched cases, 9 (45%) submitted a revised LOI to E4A, and 1 (5%) submitted their research proposal to another funder. Among the nine partnerships that submitted a revised LOI to E4A, two were invited to submit a full proposal and received funding for their projects. The partnership that submitted its research proposal to another funder was ultimately awarded a large grant. Altogether, 3 of the 10 cases (30%) that submitted a revised proposal to either E4A or another funder received funding for their projects.

**Figure 2.**
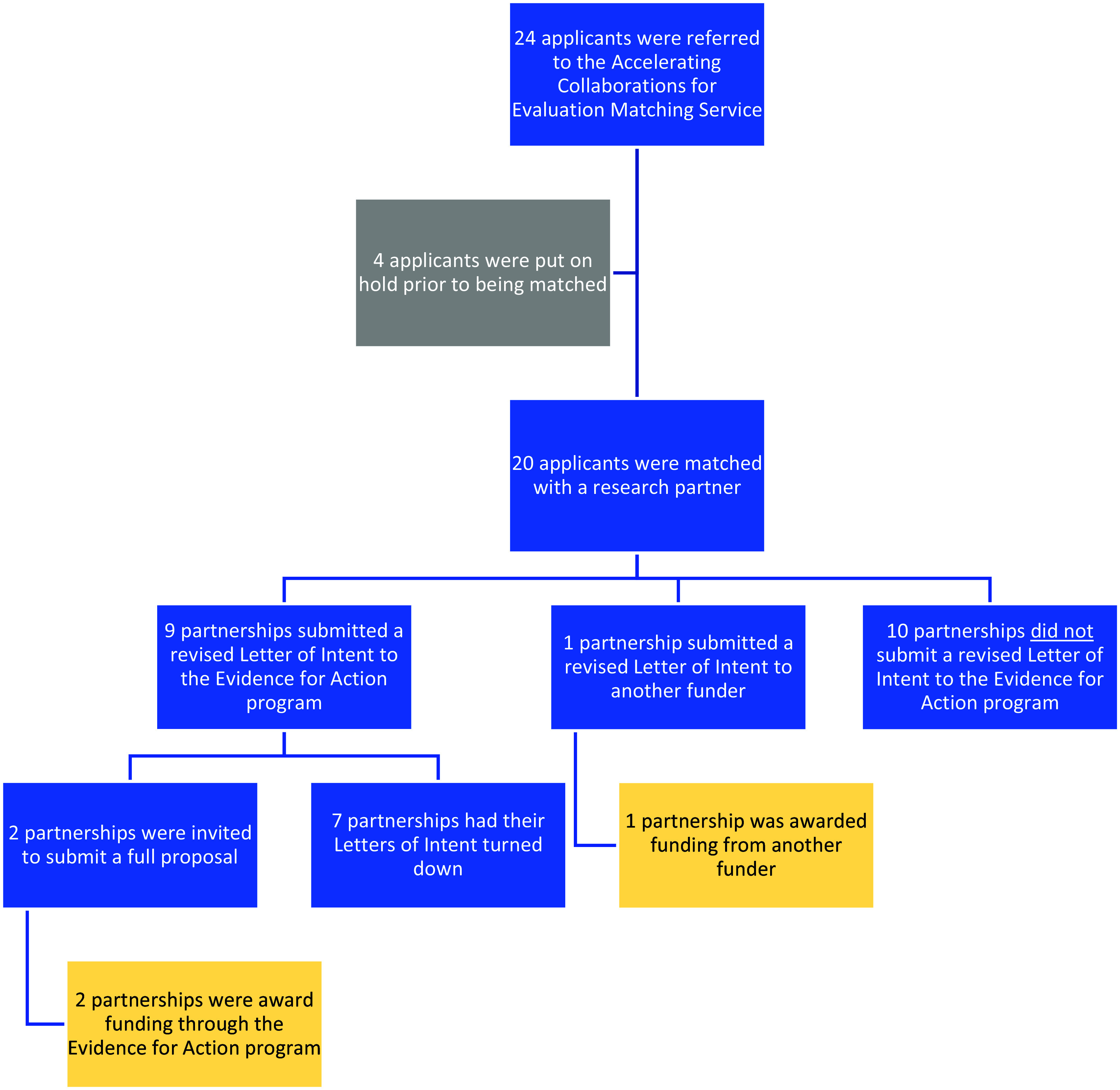
Outcomes of applicants referred to the ACE matching service.

Among the 10 matched cases that did not submit a revised application to either E4A or another funder, 1 applicant continued LOI revisions, 2 had changes in organizational leadership at the community organization that precluded their ability to submit a revision, 1 community organization closed, 1 match was lost to follow-up, 1 applicant withdrew from the matching service, and 4 decided against resubmitting their LOI because they believed it was unlikely that their revision would be accepted by E4A. See Figure 2 for a flowchart of the outcomes of the matching service.

### Utilization of seed funding

Eleven out of 20 (55%) matches requested seed funding ranging from $3,125.00 to $10,000. The most common requests for funds included reimbursement for the time spent working on the LOI revisions together (meetings, phone calls), travel expenses to meet in person (e.g., transportation, lodging, meals) (this option was eliminated during COVID-19 travel restrictions), and funding for preliminary data collection. Since most partnerships submitted their seed funding applications after revising their LOIs, those who submitted a revised LOI were more likely to request seed funding than those who did not.

### Results of the 3-, 6-, and 12-month surveys

The results from the three-month survey are displayed in Table 1. Of 40 potential respondents, we received 18 completed surveys (45%). In response to the question, “Do you consider your partnership to be a successful one?” 16 of 18 respondents said they considered their partnership a success so far, and 2 said it was too soon to say. The participants described a successful partnership as having a shared passion for the work, having the opportunity to learn about each other’s different disciplines, collaborating well together, and being inspired by their partner’s expertise, creativity, and enthusiasm. One respondent summed it up in the following way:

**Table 1.** Results of the three-month survey (*n* = 18)

Statement	% Agree/strongly agree	% Neutral/ not sure
I am satisfied with the communication I have had with my Accelerating Collaborations for Evaluation (ACE) partnership acceleration liaison (PAL).	100	0
The amount of seed funding was adequate for the needs of the partnership.	79	21
The allocation of seed funding among partners was fair.	84	16
The partner I was matched with was an appropriate collaborator for me.	100	0
My partner is meeting my expectations.	100	0
My partner is providing valuable knowledge, resources, or expertise to the project.	100	0
I am pleased with the way the collaboration is proceeding.	100	0
I believe this collaboration has/will result in an improved letter of intent/research proposal.	100	0
If our letter of intent does not result in an invitation for a full proposal, we intend to submit our proposal to another funder.	81	16
Overall, I am satisfied with the assistance that I have received from the Accelerating Collaborations for Evaluation (ACE) matching service.	94	6

I would have never met this partner without the ACE Matching Service. I’m honored they found me a partner who was interested in my work. I liked that they screened us both first before introducing us to make sure that our timing, availabilities, and interests matched. This allowed our first meeting to be fruitful and start off successful.

In response to the question, “How are you (or your organization) benefiting from this partnership?” respondents stated that it was helping them to create a data-driven evaluation of a new program, deepening their understanding of the value of their work, and helping them to structure their evaluation in a scientifically rigorous way. Additionally, one participant remarked, *“I think having the backing of ACE helped us gain legitimacy both within and outside of our organization.”*

Some of the challenges respondents reported included establishing the relationship, building trust, communicating long-distance, and coordinating multiple meeting schedules. Others mentioned that the COVID-19 pandemic caused many challenges, and one participant noted that one of their challenges was the need to change course (more than once) based on E4A feedback on the study design. One participant articulated the problems that small organizations have in developing a scientifically rigorous evaluation of their programs:

We’re a small organization with a limited budget, and it can be difficult to meet the demands of a rigorous RCT or other evaluation program. This is an ongoing challenge for us, and we tend to avoid doing serious evaluations because we simply do not have the time or capacity.

When asked if there was anything else the ACE Matching Service could do to help support their partnership, most respondents said “no” and reported being very pleased with their experience. For example, one participant commented, *“This is a remarkable program, and we are very appreciative of the support and resources that you have shared. In our opinion, the model is effective.”*

When asked if they had suggestions for improving the service, most respondents either offered none or expressed appreciation for it (e.g., *“This partnership and mentoring program have been extremely helpful to us!!!!”).* One respondent suggested that ACE *“…should consider being used for other projects that involve cross-sector collaborations or partnerships between academic institutions and the public sector.”* However, one applicant admitted being discouraged: *“By the end of the process… our team felt a bit discouraged about the potential for funding and questioned why we were picked for the matching service.”*

Out of 40 potential respondents, we received 17 completed surveys to both the 6-month and 1-year surveys (42.5%). In response to the 6-month survey, 17 (94%) respondents agreed or strongly agreed with the statement, “The partner I was matched with was an appropriate collaborator for me;” 16 (88%) agreed or strongly agreed with the statement, “I am pleased with the way the collaboration is proceeding;” and 17 (83%) agreed or strongly agreed to the item, “Overall, I am satisfied with the assistance that I have received from the ACE Matching Service.”

Among the 17 respondents to the one-year survey, 100% reported being satisfied or very satisfied with their partner; 94% were satisfied or very satisfied with the design consultation they received; and 82% were satisfied or very satisfied with the matching service.

### Qualitative findings

During the pilot, some partnerships opted not to submit revised LOIs after concluding that the evaluation design required by E4A was impractical. Others submitted LOIs but were not invited to advance to a full proposal. Even so, every partnership described the collaboration itself as valuable and rewarding. This feedback prompted us to broaden our focus to include not only funding outcomes but also the intrinsic value of each partnership.

We conducted separate interviews with 14 applicants and researchers. Twelve of these represented six matched partnerships, while the remaining two interviewees were single applicants. Overall, the interviewees included a mix of those who did (*n* =11) and did not (*n* = 3) submit a revised LOI. Ultimately, only 2 of the 14 interviewees (14%) – both part of a matched partnership – received E4A funding. Based on the feedback from our participants through the in-depth interviews, we believe that the benefits that resulted from these partnerships were the most valuable result of the matching service.

When asked about the benefits of their partnership, most participants stressed the advantages of the complementary expertise brought to the collaboration by the applicant and the researcher. The factors that facilitated the success of the partnerships were described as having a shared vision and a genuine interest in each other’s work, as well as a relationship that was trusting, open, warm, enthusiastic, and authentic. When asked to provide their own definition of the success of their partnership, some participants based it on how much they learned from their partner, others defined it as a partnership that was mutually respectful, productive, fulfilling, and enjoyable, and others described it as the potential for having a sustained collaboration. Notably, few respondents defined success as obtaining funding for their project. The offering of seed funding meant a great deal to both applicants and researchers and signaled that this was a genuine endeavor worthy of E4A’s financial support.

The most frequently cited obstacles were time constraints and competing demands. Both academic researchers and community partners struggled to balance work on the LOI revision with their other professional responsibilities.

The second most commonly reported challenge was meeting the expectations of the E4A program. After design consultations with the E4A liaison, several applicants realized that their projects lacked a sufficient sample size or an appropriate comparison group. Recognizing that their study design would not meet the program’s methodological requirements, some applicants ultimately chose not to submit a revised LOI.

Another frequently cited barrier was the reduction or loss of funding for the intervention. Since E4A only funds evaluations, any cuts to the intervention funding jeopardized the evaluation. In several cases, applicants lost – or saw reductions in – their intervention funding after being matched with a research partner, which significantly impacted the work they had planned to pursue together.

Overall, most participants spoke highly of the ACE Matching Service. A handful of participants noted that they believe this program plays a critical role in helping to connect community-based organizations to the expertise needed to develop competitive proposals for submissions to funders like RWJF. Others remarked that researcher partners provided greater access to professional networks and funders that community-based organizations either hadn’t previously known about or hadn’t previously accessed.

A few participants expressed a need for more information about the matching service. They suggested offering webinars or virtual meetings to provide reminders about the service’s purpose, partnership expectations (e.g., building fair and equitable partnerships), the roles of key stakeholders (ACE, PALs, E4A, RWJF), and timelines. They also recommended access to an online platform or “toolkit” with these resources available throughout the matching process. While participants recalled learning much of this information early on, they wanted it to be accessible at other stages of the experience. One participant noted the value of preparing for “bumps in the road,” including strategies for addressing challenges and acknowledging that outcomes might not always meet expectations. Another participant expressed interest in the opportunity to connect with other matched partners to share insights about partnerships and seed funding.

While the matching service received much praise, some participants noted E4A’s strong emphasis on randomized controlled trials (RCTs) and the difficulty many organizations face in designing and conducting rigorous RCTs for programs. Another participant observed that community-based organizations might perceive themselves as unlikely to compete successfully for an RWJF grant and, as a result, may choose not to apply.

Some participants expressed a desire for greater transparency about the competitiveness of E4A applications. They felt there was insufficient clarity regarding the criteria for success and what it takes to submit a fundable application to RWJF. Even after attending design consultations and collaborating with a research expert to develop an evaluation plan, participants found it extremely challenging to meet the level of rigor expected by E4A. Table 2 contains a selection of representative quotes from the interviews.

**Table 2.** Representative quotes from the in-depth interviews

Theme	Quotes from applicants	Quotes from researchers
Benefiting from the Accelerating Collaborations for Evaluation (ACE) Match	“The benefit is that we were able to write a much stronger proposal. [The researcher] brought just what I needed to the submission process, in the sense of increasing the methodological rigor of the proposal.”	“So, it’s kind of a two-way street. I’m learning about his world … and he’s learning what’s involved in a high-quality, rigorous evaluation. So, it’s a little bit of cross-pollination, which is very nice.”
Moving the Accelerating Collaborations for Evaluation (ACE) Partnership Forward	“The nice thing about working with [researcher’s name] … was that she was really easy to work with. Very receptive to this whole idea. Her enthusiasm for the project was really, really infectious.” “It really helped me along knowing that she believed in it; she thought it was a wonderful program.”	“I think the fact that [the applicant] and I both entered into this partnership with enthusiasm and openness… I have to give [the applicant] credit for his willingness to hand over a part of his baby, the evaluation part, to somebody else, and trust that they would do well by him…”
Defining a Successful Match	“What has come out of our collaboration has been an authentic relationship…that will last beyond this opportunity;” “I would define it as being excited and eager to continue working together.”	“I think the success of the partnership could be defined by what we learned about each other’s disciplines.”
Impact of Seed Funding	“It felt good to me knowing that ACE was willing to make that kind of investment in our proposal, so that kind of strengthened our commitment to doing it well.”	“I was just astounded that it was available because I’ve been doing research for 30 years almost now, and I’ve never seen seed funding…To me, it showed that it was serious…So the fact that you said, here’s some seed funding, use it to get to know each other, I think was really profound and very important.”
Challenges or Barriers to the Accelerating Collaborations for Evaluation (ACE) Partnership	“We’re all grant-funded, and I run a department with 20 people. So, my priority has to be fundraising and getting money.”	“I think we’re all often maxed out and have little bandwidth even though something is really important. And I’m personally always putting out fires and pulled in a thousand directions. So, to me, that’s the biggest barrier.”
Overall Value of the Accelerating Collaborations for Evaluation (ACE) Matching Service	“It’s just a phenomenal idea to me. It is one way in which Robert Wood Johnson is, and other, I guess, organizations, are helping organizations to level up their capability. It’s a brilliant, brilliant idea. It’s a beautiful start in a good direction. But certainly, there’s a lot more to do.”	“… I think this is kind of a social equity matching. Like, I think you’re helping level up who is able to engage with some of these funders and processes. So, I think it’s really valuable.” “I’m a huge fan of this program and I wish more people would set something like this up.”

## Discussion

This pilot project tested a matching service model aimed at helping community applicants secure funding to evaluate interventions that enhance health and health equity. Although this was a worthy goal, its feasibility was untested. From the outset of the project, neither E4A nor ACE could predict how many cases would be referred to the matching service, the time required to find appropriate matches for applicants, how long partnerships would take to revise their LOIs, the amount for seed funding requests, or the outcomes of the revised submissions.

The COVID-19 pandemic emerged midway through the pilot and introduced significant challenges and delays. Fewer LOIs were submitted to E4A, reducing the pool of potential referrals. Researchers and applicants from community organizations faced additional personal and professional pressures, complicating the matchmaking process and often pulling matched partners away from revising their LOIs. In some cases, interventions stalled or were discontinued altogether.

Despite these challenges, the ACE team concentrated efforts on increasing the number of resubmissions and funded projects. To date, two projects received grant awards from E4A – a rate comparable to the overall funding rate for all applicants to the program at that time – and one received funding through a different funder. While some matches did not result in funding, applicants and researchers considered their partnerships highly valuable, often describing these collaborations as unique opportunities to connect with partners they might not have met otherwise.

### Lessons learned

Several important lessons emerged from the pilot. First, the specificity of the needs of each applicant underscored the importance of a personalized matchmaking process. While a database of willing academic partners was initially considered, it became evident that successful matches required the careful attention of a PAL who could identify and vet researchers capable of meeting applicants’ unique needs and preferences. The role of the PAL was essential in eliciting and clarifying applicant needs, selecting suitable researchers, and fostering mutually beneficial partnerships.

Second, the flexibility of the matching service proved critical. While the original plan allowed for a single design consultation per partnership, the utility of this service led to more of these sessions, with most partnerships participating in two or three design consultations before submitting their revisions. Similarly, while seed funding was highly valuable to some participants, others found the application process burdensome or unnecessary. This insight prompted a reevaluation of the seed funding process to make financial support more accessible.

Third, over the course of the pilot study, the E4A program recognized that the standard of rigor required for LOIs to be selected to receive funding was beyond what some community organizations could achieve. For example, in some cases, they were not able to obtain a large enough sample size, or able to obtain an appropriate comparison group. Consequently, E4A has revised its selection criteria to be more consistent with the reality of community research endeavors.

While the RWJF created this matching service to improve the capacity for the funding of CEnR, many institutions around the country fund community-academic partnerships (CAPs) through the Clinical and Translational Science Awards program (https://ncats.nih.gov/research/research-activities/ctsa). Even though the ACE Matching Service model and CAPs may differ in how partnerships are formed, the types of research projects supported, and the expected evaluation designs, both approaches demonstrate the value of seed funding, TA, and complementary expertise in building successful partnerships [19].

Respect and trust are consistently cited as the primary drivers of successful CAPs [20,21], supported by clear communication, shared goals, and joint decision-making [22]. Tang further stresses that partnerships must be both meaningful and equitable to thrive [23], and Woolford demonstrates that partners need time – preferably face to face – to work and socialize together in order to deepen these bonds [24]. Our findings align closely with this body of evidence. Moreover, we structured our seed funding to compensate community and academic partners equally and encouraged in-person meetings, often over shared meals. Several teams used their funds specifically to nurture the partnership in this way.

We found one study by Ramirez that specifically examines the development and evaluation of a matching service connecting community organizations and academic researchers [25]. Similar to our findings, this study reveals that trust, mutual benefit, and effective communication are key to successful partnerships. It also notes that while the matchmaking process accelerated initial connections, it was not able to replace the foundational need for building partnerships rooted in mutual trust and respect.

The reported success of the ACE Matching Service prompted RWJF to renew funding for the program, which is currently active. Then, in 2022, RWJF launched the Health Equity Scholars for Action (HES4A) program, an initiative aimed at advancing the academic careers and research goals of health equity scholars. At this point, ACE was invited to establish another matching service, which involved matching the program’s scholars with mentors and career coaches to help them advance toward tenure. Now in its second cohort, this initiative demonstrates the continued relevance and adaptability of the ACE model in fostering impactful partnerships.

## Limitations

There are some limitations to this study. Getting all of our participants (both applicants and researchers) to respond to our surveys was challenging, as their primary focus was revising their LOIs. Additionally, our request for survey participation coincided with the height of the COVID-19 pandemic, when many community practitioners and public health researchers faced COVID-19-related constraints. As a result, participation in the 3- to 6-month surveys and in-depth interviews was incomplete. However, we believe these concerns are mitigated by our collection of both qualitative and quantitative data and our first-hand experiences and interactions with participants, which provided a comprehensive range of positive and negative feedback about the matching service. Because we did not follow our participants beyond the evaluation reporting, we are unaware of the long-term status of the partnerships.

## Conclusions

We successfully developed a matching service that fostered valuable partnerships between E4A applicants and their matched researchers. The service’s core value lies in connecting individuals unlikely to collaborate otherwise and supporting mutually beneficial partnerships. It also enhanced the capacity of nonacademic practitioners by providing skills, connections, networking opportunities, and the confidence to submit applications to other funders. We believe this model can be adopted by others seeking to create research partnerships to build capacity for the evaluation of community initiatives.
